# Supplementary material for: Association between oral microbiome and seven types of cancers in East Asian population: a two-sample Mendelian randomization analysis
Source: Front Mol Biosci. 2023 Nov 21;10:1327893. doi: 10.3389/fmolb.2023.1327893 (PMC10702768; doi:10.3389/fmolb.2023.1327893)
Supplement: Supplementary file 1 [file DataSheet1.ZIP › Supplementary Materials/MR plots of saliva/saliva═╝/colorectal cancer/pheno.899_to_colorectal cancer_forest.pdf]

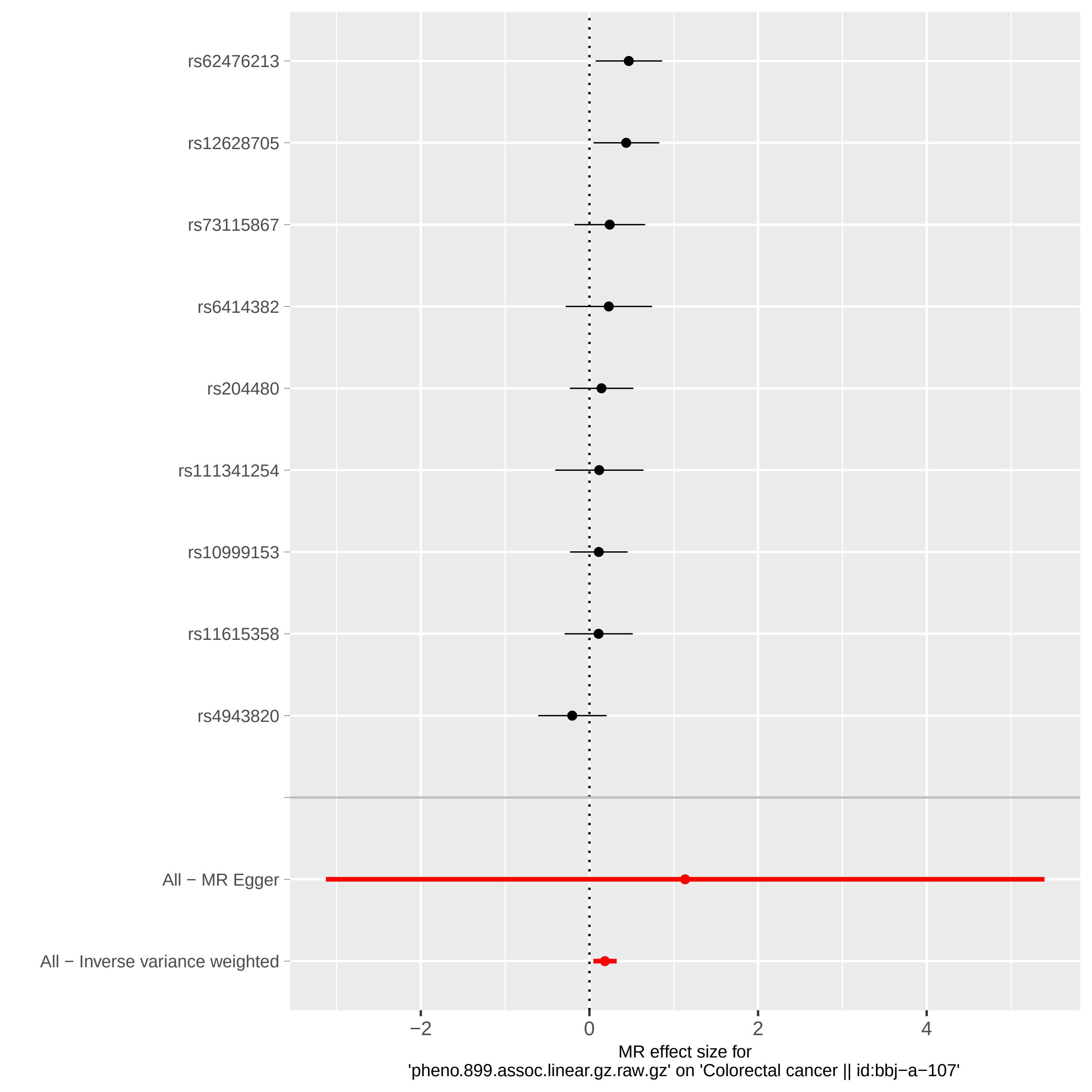

rs62476213
rs12628705
rs73115867
rs6414382
rs204480
rs111341254
rs10999153
rs11615358
rs4943820
All − MR Egger
All − Inverse variance weighted
−2
0
2
4
MR effect size for
'pheno.899.assoc.linear.gz.raw.gz' on 'Colorectal cancer || id:bbj−a−107'
